# Lesion Topography and Clinical Features Associated With Respiratory Failure in Patients With Medullary Infarction

**DOI:** 10.1002/brb3.70259

**Published:** 2025-01-08

**Authors:** Na Zhao, Ran Liu, Yajing Zhang, Ling Ling, Chao Zhang, Ting Zhang, Wei Yue

**Affiliations:** ^1^ Department of Neurology, Tianjin Key Laboratory of Cerebrovascular and Neurodegenerative Diseases, Clinical College of Neurology, Neurosurgery and Neurorehabilitation, Tianjin Huanhu Hospital Tianjin Medical University Tianjin China

**Keywords:** clinical characteristics, lateral medullary infarction, lesion pattern, medial medullary infarction, respiratory failure

## Abstract

**Background:**

Respirator failure (RF) is a severe malignant complication in both lateral medullary infarction (LMI) and medial medullary infarction (MMI) patients. However, the differences in clinical and radiological manifestations associated with RF between patients with LMI and MMI have not been fully elucidated.

**Methods:**

A total of 435 consecutive patients with MI within 7 days of onset in our institute were retrospectively enrolled from January 2017 to January 2024. Lesions were categorized both rostral‐caudally and horizontally, and clinical characteristics were collected to evaluate the correlation between them and RF that occurred within 10 days of stroke onset.

**Results:**

Among the 435 patients, 33 patients developed RF, with 19 exhibiting LMI and 12 exhibiting MMI. Multisegment involvement was more common among LMI patients experiencing RF compared to those without (52.6% vs. 23.9%, *p* = 0.012). However, this difference was not observed among MMI patients. Large (*n* = 12, 63.2%, *p* = 0.014) and typical (*n* = 6, 31.6%, *p* = 0.016) lesions were more common among LMI patients with RF. In MMI patients with RF, nine (75.0%) patients had long lesions extending from the ventral to the dorsal medulla, with six of these cases involving bilateral lesions, as V‐shape or heart‐shape on MRI. Univariate analysis of clinical symptoms revealed that bulbar symptoms, urinary retention, and pulmonary infection were significantly more common in both the LMI and MMI groups with RF.

**Conclusions:**

Specific lesion patterns, such as large and typical infarctions in LMI patients or long lesions, particularly those with bilateral infarctions, as V‐shape or heart‐shape on MRI in MMI patients, appear to correlate with a higher incidence of RF, while clinical symptoms associated with RF are similar in LMI and MMI.

## Graphical abstract

This study aims to clarify the lesion patterns and clinical characteristics of medullary infarction (MI) patients associated with respiratory failure (RF) and assess their differences between lateral medullary infarction (LMI) and medial medullary infarction (MMI). Specific lesion patterns, such as large and typical infarctions in LMI patients or long lesions, particularly those with bilateral infarctions, as V‐shape or heart‐shape on MRI in MMI patients, appear to correlate with higher incidence of RF.

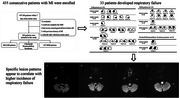

## 1 Introduction

Medullary infarction (MI), as a relatively rare condition, accounts for approximately 7.0% of posterior circulation ischemic strokes (Sciacca et al. 2019). While the overall prognosis is favorable, there have been several reported cases of respiratory failure (RF), which can exacerbate the clinical course and lead to sudden, unforeseen death (Norrving and Cronqvist 1991; Zhang et al. 2021). The network of neurons in the medulla oblongata that controls respiration includes the dorsal respiratory group (DRG) and ventral respiratory group (VRG). These two groups control the respiratory rhythm (Ciumas, Rheims, and Ryvlin 2022). Current research on MI complicated by RF has primarily focused on lateral medullary infarction (LMI). Studies indicate that about 2%–6% of patients with pure LMI suffer from RF during the course of the disease (J. Kim 2003; Ogawa et al. 2015), and severe bulbar symptoms, urinary retention, prestroke history, and older age were related to RF (Pavsic et al. 2021; Saito et al. 2022). Currently, there is a lack of large‐scale studies addressing the incidence of RF in patients with medial medullary infarction (MMI), while whether there are disparities in clinical characteristics between LMI and MMI patients who had RF remains unclear. Moreover, few research have explored specific lesion patterns that may indicate a higher incidence of RF during the course in LMI and MMI patients. Therefore, this study aims to clarify the lesion patterns and clinical characteristics of MI patients associated with RF and assess their differences between LMI and MMI.

## 2 Materials and Methods

### 2.1 Participants

Consecutive patients with MI confirmed by magnetic resonance imaging (MRI) within 7 days of onset in Tianjin Huanhu Hospital were retrospectively enrolled from January 2017 to January 2024. All patients underwent complete cerebrovascular evaluation, including color Doppler ultrasound + magnetic resonance angiography (MRA), computed tomography angiography (CTA), or digital subtraction angiography (DSA). Exclusion criteria were as follows: (1) patients with contraindications or other reasons could not complete the MRI examination; (2) patients with inflammation, tumor, hemorrhage, demyelination, and other bulbar lesions; (3) severe neurological deficits due to previous stroke or other diseases; and (4) patients with previous history of MI.

### 2.2 Data Collection and Definitions

Demographic data, cerebrovascular risk factors, medical history, clinical symptoms, and signs were collected from the medical records of screened patients. RF was defined as a condition in which life support could not be achieved by normal oxygen administration alone because of the sudden‐onset respiratory rhythm abnormalities or a decrease in blood oxygen saturation and where mechanical ventilation was required (Kumral et al. 2011). RF secondary to pulmonary infection was excluded. Mechanical ventilation support was required for more than 24 h. Neurological symptoms included those observed upon admission and those developed after stroke onset. Severe dysphagia was defined as a patient scoring 5 points on the Water Swallowing Test (Jang and Kim 2021). Major complications during hospitalization included urinary retention, pulmonary infection, and stress ulcer. For patients complicated with RF, the incidence of these above complications was only assessed before the occurrence of RF to avoid confounding effects due to RF. We calculated the days from stroke onset to the occurrence of RF. The clinical outcome was evaluated by means of the modified Rankin scale (mRS) score at 90 days, which was classified as favorable (mRS score, 0–2) or poor (mRS score, 3–6).

### 2.3 Distribution of Lesions on DWI

The location of infarcts was determined using diffusion‐weighted images (DWIs) that were acquired upon admission or during hospitalization. DWIs were obtained using 3.0 T MRI and acquired in the axial plane with *b* values of 0 and 1000 s/mm^2^. Two neurologists who were blinded to clinical data assessed all MRI scans. Infarctions within the medulla were categorized into LMI and MMI based on arterial blood supply. LMI was mainly supplied by the posterior spinal artery and posterior inferior cerebellar artery (PICA), while MMI was mainly supplied by the anterior spinal artery and the medullary branches of the vertebral artery. Reinhold hemimedullary syndrome (RHS) was defined as simultaneous involvement of both the medial and lateral medulla.

Lesions detected by DWI were categorized both rostral‐caudally and horizontally according to the classification by J. Kim (2003). Rostral‐caudal classification included four groups: (1) rostral medulla, extending from pontomedullary sulcus to inferior cerebellar peduncle; (2) middle medulla, extending from inferior cerebellar peduncle to inferior olivary nucleus; (3) caudal medulla, extending from inferior olivary nucleus to foramen magnum; and (4) multisegments, involvement of two or more segments from the previous three groups.

The horizontal classification for LMI lesions included the following (J. Kim 2003): typical: band‐shaped lesions mainly involving the dorsolateral portion; ventral: similarly shaped lesions locating more ventrally and part of the inferior olive was involved; large: large lesions extending ventrally and affecting the majority of the dorsolateral area; dorsal: lesions limiting to the most dorsal or dorsolateral region; and lateral: superficial lesions confining to the lateral without extending dorsally.

In MMI, lesions were classified ventro‐dorsally into three groups as follows (J. Kim and Han 2009): ventral (V), mainly containing the corticospinal tract; middle (M), mainly containing the medial lemniscus; and dorsal (D), mainly containing the medial longitudinal fasciculus. In addition, we created three axial grades depending on the segments of V, M, and D as follows: short lesion, only one segment was involved (V/M/D); middle lesion, two segments were involved (V + M, M + D); long lesion, three segments were involved (V + M +D).

Vascular lesions were evaluated using color Doppler ultrasound + MRA, CTA, or DSA. Presumed stroke mechanisms were defined according to the “Trial of ORG 10172 in Acute Stroke Treatment” criteria based on the aforementioned cerebrovascular imaging (Adams et al. 1993). Vertebral artery dissection was categorized as “other determined etiology” and defined when vascular imaging indicated arterial dissection with concurrent neck/occipital pain.

### 2.4 Statistical Analysis

All statistical analyses were performed using SPSS 26.0 statistical software. Univariate analysis was performed to compare the clinical and radiological characteristics between patients with or without RF. Independent *t*‐test and Wilcoxon signed rank test were used to analyze continuous variables that were expressed as medians ± interquartile ranges (IQRs) or mean ± standard deviation. Pearson's chi‐square test and Fisher's exact test were used to analyze categorical variables that were presented as numbers and percentages. *p* values < 0.05 were considered statistically significant.

## 3 Results

**FIGURE 1:**
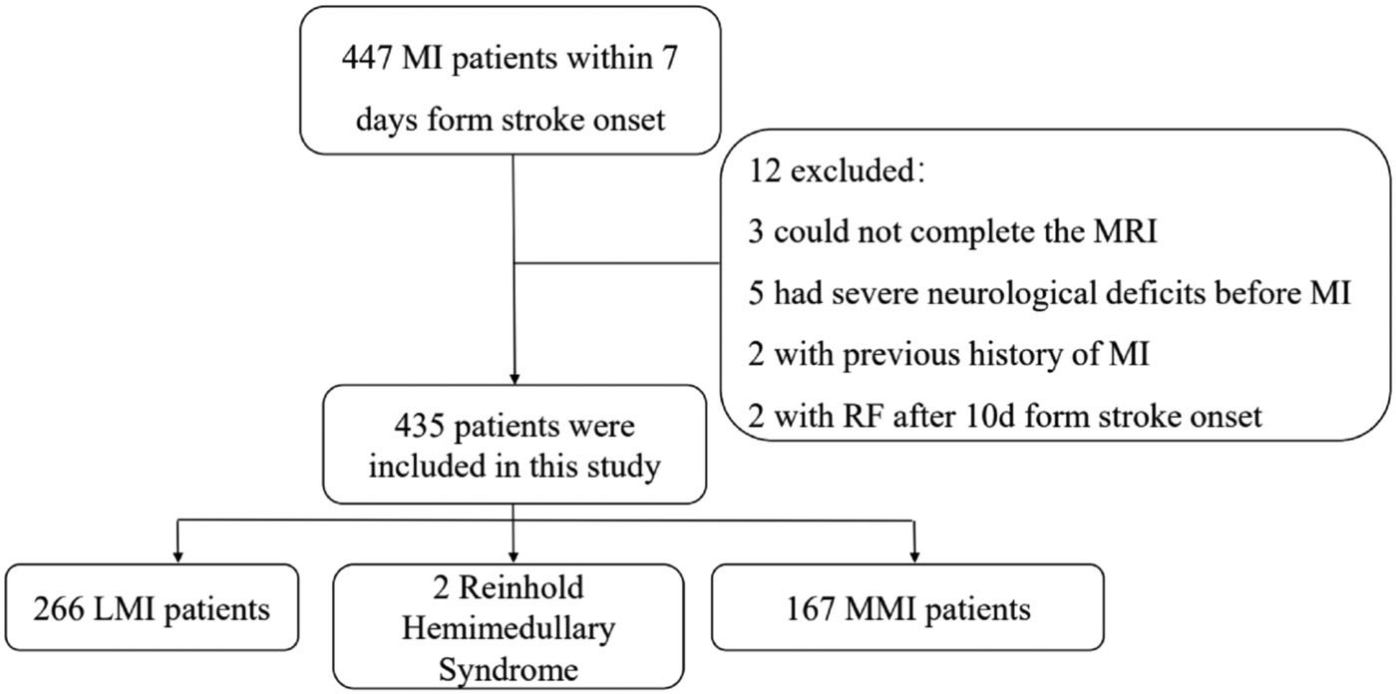
The flowchart of patient selection. LMI, lateral medullary infarction; MI, medullary infarction; MMI, medial medullary infarction; RF, respiratory failure.

A total of 435 patients were enrolled in this study, including 360 males (82.8%) and 75 females (17.2%), aged 61 (±14) years. The flowchart of patient selection is depicted in Figure 1. Their stroke risk factors and clinical characteristics are shown in Table 1. Among the enrolled patients, 33 (7.6%) developed RF during the course of the disease. There were no significant differences observed in clinical characteristics such as gender and past medical history between patients with and without RF. However, patients who developed RF were younger (*p* = 0.022) and had higher systolic blood pressure on admission (*p* = 0.014). In addition, the stroke classification differed between patients with and without RF (*p* = 0.024), primarily due to the higher incidence of large artery atherosclerosis (LAA) in the former group.

**TABLE 1:** The baseline characteristics.

Variables	Total (*n* = 435)	RF (*n* = 33)	Non‐RF (*n* = 402)	*p* value
Age, years	61 (±14)	60 (±14)	63.7 (±11.29)^a^	**0.022**
Gender, *n* (%)				
Male, *n* (%)	360 (82.8)	27(81.8)	333 (82.8)	0.814
Female, *n* (%)	75 (17.2)	6 (18.2)	69 (17.2)	
Hypertension, *n* (%)	354 (81.4)	24(72.7)	330 (82.1)	0.241
Diabetes, *n* (%)	198 (45.5)	13 (39.4)	185 (46.2)	0.586
Cardiac diseases, *n* (%)	106 (24.4)	7(21.2)	99(24.6)	0.833
Atrial fibrillation, *n* (%)	19 (4.4)	2(5.7)	17(4.2)	0.647
Dyslipidemia, *n* (%)	190 (43.7)	13(39.4)	177(44.0)	0.716
Stroke, *n* (%)	124 (28.5)	14 (42.2)	110(27.4)	0.073
Smoking, *n* (%)	236 (54.3)	16(48.5)	220 (54.7)	0.586
Drinking, *n* (%)	131 (30.1)	11 (33.3)	120 (29.9)	0.695
SBP at admission, mmHg	145 (±31)	146 (±31)	139.03 (±19.47)^a^	**0.014**
DBP at admission, mmHg	87 (±18)	87 (±18)	84.45 (±12.50)^a^	0.263
Serum glucose at admission, mmol/L	5.61(±2.79)	5.64(±2.74)	5.04(±2.80)	0.054
LDL‐C at admission, mmol/L	2.94 (±1.07)	2.95 (±0.90)	2.76 (±0.99)	0.211
**Stoke subtype by TOAST**				
Large‐artery atherosclerosis	282 (64.8)	30 (90.9)	252 (62.7)	**0.024**
Cardioembolism	14 (3.2)	1 (3.0)	13 (3.2)	
Small‐artery occlusion	114(26.2)	2 (6.1)	112(27.9)	
Other etiology	5 (1.1)	0 (0)	5 (1.2)	
Undetermined	20 (4.6)	0 (0)	20 (5.0)	

^a^ Continuous variables which coincided with normal distribution were expressed as mean ± standard deviation.

Abbreviations: Non‐RF, no respiratory failure; RF, respiratory failure. Bold values indicate *p* < 0.05.

**FIGURE 2:**
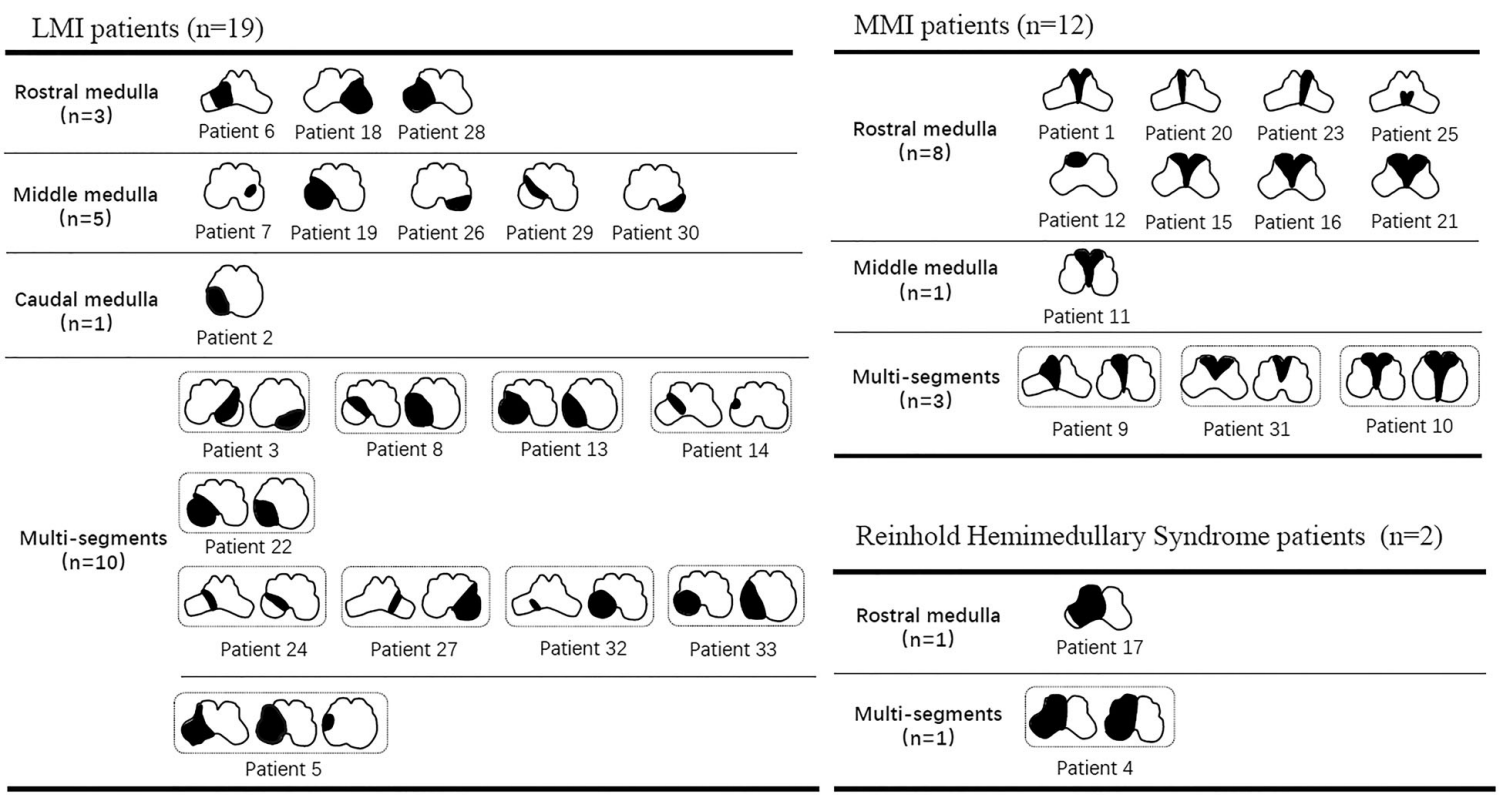
Lesion topography of medullary infarction with respiratory failure during the course of the disease.

Among the 435 patients, 266 exhibited LMI, with 19 (7.1%) experiencing RF, while 167 displayed MMI, with 12 (7.2%) experiencing RF. Two patients presented with RHS, both of whom developed RF during the course of the disease. The lesion topography is illustrated in Figure 2.

**FIGURE 3:**
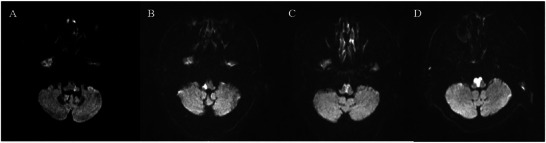
Specific lesion patterns in medullary infarction with respiratory failure on DWI. (A) Typical in LMI, (B) large in LMI, (C) Y‐shaped in MMI, and (D) heart‐shaped in MMI.

Among 19 LMI patients with RF, 13 had right‐sided medullary infarcts and 6 had left‐sided medullary infarcts. There was no significant difference in the distribution of infarcts between LMI patients with RF and those without (*p* = 0.237) (Table 2). However, among the 12 MMI patients with RF, 2 had right‐sided infarcts, 2 had left‐sided infarcts, and 8 had bilateral infarcts, which demonstrated significant differences in the distribution of infarcts between the three groups (*p* < 0.001) (Table 3). In rostro‐caudal subtypes on MRI, multisegment involvement was more common among LMI patients experiencing RF compared to those without (52.6% vs. 23.9%, *p* = 0.012). However, this difference was not observed among MMI patients (Tables 2 and 3). Further analysis of single‐segment involvement revealed that MMI patients with RF had higher proportions of rostral involvement compared to those without (88.9% vs. 72.9%). However, there were no statistical differences between those two groups (*p* = 0.551). No such differences were observed in LMI patients (*p* = 0.828) (Tables 2 and 3). In horizontal subtypes, large (*n* = 12, 63.2%, *p* = 0.014) and typical (*n* = 6, 31.6%, *p* = 0.016) lesions were more common among LMI patients with RF, and they were also the predominant types in this group (Table 2). Among MMI patients with RF, nine (75.0%) patients had long lesions extending from the ventral to the dorsal medulla, one case had a middle lesion (8.3%), and two cases had short lesions (16.7%), with long lesions being more frequent among MMI patients with RF (*p* = 0.010) (Table 3). Further analysis of the imaging characteristics of the 12 MMI patients with RF revealed that 6 (50.0%) had bilateral involvement with long lesions, manifesting as Y‐shaped or heart‐shaped infarctions on MRI (Figure 3).

**TABLE 2:** Magnetic resonance imaging findings in patients with LMI.

Variables	RF (*n* = 19)	Non‐RF (*n* = 247)	*p* value
Right side lesion	13 (68.4)	131 (53.0)	0.237
Left side lesion	6 (31.6)	116 (47.0)	
**Rostro‐caudal subtypes**			
Single segment	9 (47.4)	188 (76.1)	**0.012**
Multisegments	10 (52.6)	59 (23.9)	
Rostral^a^	3 (33.3)	54 (28.7)	0.828
Middle^a^	5 (55.6)	98 (52.1)	
Caudal^a^	1 (11.1)	36 (19.2)	
**Horizontal subtypes**			
Typical	6 (31.6)	26 (10.5)	**0.016**
Large	12 (63.2)	84 (34.0)	**0.014**
Ventral	2 (10.5)	39(15.8)	0.747
Lateral	3 (15.8)	55(22.3)	0.773
Dorsal	2 (10.5)	55 (22.3)	0.382

^a^ Patients with single segment involvement were included. Bold values indicate *p* < 0.05.

**TABLE 3:** Magnetic resonance imaging findings in patients with MMI.

Variables	RF (*n* = 12)	Non‐RF (*n* = 155)	*p* value
Right side lesion	2 (16.7)	73 (47.1)	**< 0.001**
Left side lesion	2 (16.7)	75 (48.4)	
Bilateral lesions	8 (66.6)	7 (4.5)	
**Rostro‐caudal subtypes**			
Single segment	9 (75.0)	129 (83.2)	0.439
More segments	3 (25.0)	26 (16.8)	
Rostral^a^	8 (88.9)	94 (72.9)	0.551
Middle^a^	1 (11.1)	30 (23.2)	
Caudal^a^	0 (0)	5 (3.9)	
**Horizontal subtypes**			
Short lesion	2 (16.7)	66 (42.6)	**0.010**
Middle lesion	1 (8.3)	40 (25.8)	
Long lesion	9 (75.0)	49 (31.6)	

^a^ Patients with single segment involvement were included. Bold values indicate *p* < 0.05.

The patients' neurological symptoms/signs are summarized in Table 4. The symptoms/signs were divided into very common (∼50%) and less common (< 40%). In patients with LMI and MMI, the same very common symptoms/signs were sensory symptoms, dizziness or vertigo, and nausea. In LMI patients, very common symptoms/signs also included gait ataxia and severe dysphagia, while in MMI patients, very common symptoms/signs included dysarthria and hemiparesis.

**TABLE 4:** Univariate analysis of clinical symptoms in patients with LMI and MMI.

	LMI (*n* = 266)			MMI (*n* = 167)		
Variables	RF (*n* = 19)	Non‐RF (*n* = 247)	*p* value	RF (*n* = 12)	Non‐RF (*n* = 155)	*p* value
Sensory symptoms	9 (31.6)	133 (53.8)	0.638	5 (41.7)	96 (61.9)	0.222
Gait ataxia	12 (63.1)	145 (58.7)	0.811	2 (16.7)	25 (16.1)	1.000
Dizziness or vertigo	17(89.5)	213 (86.2)	1.000	9(75.0)	106 (68.4)	0.756
Horner's syndrome	2 (10.5)	72 (29.1)	0.110	1 (8.3)	7 (4.5)	0.457
Severe dysphagia	18(94.7)	126 (51.0)	**< 0.001**	10(83.3)	41 (26.5)	**< 0.001**
Dysarthria	13 (68.4)	94 (38.1)	**0.014**	11 (91.7)	90 (58.1)	**0.029**
Nystagmus	6(31.6)	71(28.7)	0.796	4(33.3)	41(26.5)	0.736
Diplopia	1(5.3)	44 (17.8)	0.214	2(16.7)	20(12.9)	0.660
Nausea	10(52.6)	153 (61.9)	0.468	4(33.3)	63(40.6)	0.764
Headache	0 (0)	15 (6.1)	0.610	1 (8.3)	11(7.1)	1.000
Hiccups	2 (10.5)	39 (15.8)	0.747	2(16.7)	5(3.2)	0.081
Facial palsy	5 (26.3)	66 (26.7)	1.000	6 (50.0)	60 (38.7)	0.543
Lingual paralysis	4 (21.1)	44 (17.8)	0.757	6 (50.0)	62 (40.0)	0.551
Hemiparesis	2 (10.5)	64 (25.9)	0.173	11 (91.7)	129 (83.2)	0.693
**Complication**						
Urinary retention	7 (36.8)	35 (14.2)	**0.017**	4(33.3)	16 (10.3)	**0.040**
Stress ulcer	5 (26.3)	58 (23.5)	0.782	4(33.3)	21 (13.5)	0.084
Pulmonary infection	11 (57.9)	54 (21.8)	**< 0.001**	10 (83.3)	20 (12.9)	**< 0.001**

Bold values indicate *p* < 0.05.

Univariate analysis of neurological symptoms revealed that severe dysphagia (90.9% vs. 41.5%, *p* < 0.001) and dysarthria (75.8% vs. 45.8%, *p* = 0.001) were significantly more common in patients with RF compared to those without in both LMI and MMI groups. Among the three complications included in this study, urinary retention and pulmonary infection showed significant differences between the two groups. However, stress ulceration did not exhibit a significant difference between them.

**FIGURE 4:**
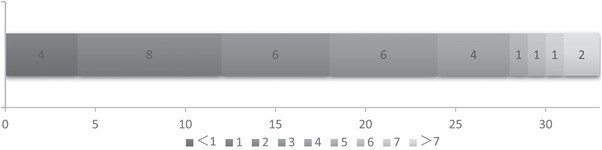
The time from stroke onset to the occurrence of RF (days). The numbers below the diagram mean the days from stroke onset to the occurrence of RF. The numbers on the diagram mean the number of patients with RF.

**FIGURE 5:**
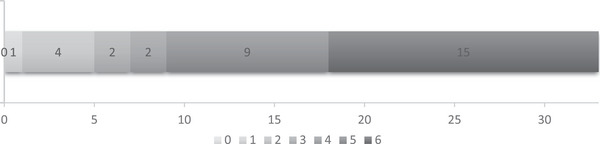
The clinical outcome at 90 days of patients with RF The numbers below the diagram mean mRS score (0–6) The numbers on the diagram mean the number of patients with RF.

Among the 33 patients with RF, the time from stroke onset to the occurrence of RF is shown in Figure 4, with 24 patients (72.7%) developing RF within 3 days after onset and 9 patients (27.3%) developing it later. The clinical outcomes at 90 days of patients with RF are presented in Figure 5. Among them, 5 patients (15.2%) had favorable outcomes, while 28 patients (84.8%) were poor, with 15 patients (45.5%) ultimately dying.

## 4 Discussion

Our study revealed that RF was a severe malignant complication in both LMI and MMI patients. Younger age, elevated systolic blood pressure on admission, and high percentage of LAA were notably prevalent in MI patients who had RF compared to those who did not. More segments of involvement were more frequently observed in patients with RF within groups of rostral‐caudal classification. Notably, the rostral medulla appeared to be predominantly affected in these cases. Within the horizontal classification, the predominant imaging pattern among LMI patients with concomitant RF was typical and large, whereas in MMI patients, it manifested as bilateral long infarctions, presenting as a V‐shaped or heart‐shaped pattern on MRI. Furthermore, severe dysphagia, dysarthria, urinary retention, and pulmonary infections exhibited significantly higher prevalence among these LMI and MMI patients during the initial 10 days after stroke onset. Respiratory complications in MI patients typically develop early in the disease course and entail a poorer functional outcome.

The previous studies have suggested that older age independently correlates with RF in pure LMI patients (Pavsic et al. 2021; Saito et al. 2022), while we did not observe a correlation between advanced age and RF. On the contrary, younger patients exhibited a higher incidence for RF during the disease. However, in our study, despite the fact that MI patients with RF were younger, this group had higher blood pressure at admission and a greater proportion of LAA. Previous research has confirmed that, compared to other stroke mechanisms, LAA was independently associated with poor outcomes in both LMI and MMI patients (Makita et al. 2019). LAA and elevated systolic blood pressure were potentially associated with inadequate cerebral perfusion compensation, which in turn was linked to poorer stroke outcomes (Lee et al. 2012). In our study, there was no significant age difference between the two groups (60.0 vs. 63.7). We hypothesize that the impact of age on respiratory outcomes may be influenced by blood pressure at admission and the proportion of LAA. It was reasonable to consider that these three factors may collectively affect respiratory outcomes in MI patients. Therefore, further analyses were needed to explore the interactions among these variables.

Previous studies have consistently demonstrated that LMI was a significant radiological feature associated with the development of RF during the course of MI (Makita et al. 2019; Mishina et al. 2014). The medullary respiratory central pattern generator is comprised of the DRG and VRG (Ciumas, Rheims, and Ryvlin 2022). In murine studies, it has been confirmed that most respiratory neurons were located around the nucleus ambiguus and the nucleus retroambigualis, corresponding to VRG, while a few respiratory neurons were identified around the ventrolateral nucleus of the solitary tract, corresponding to DRG (Ezure, Manabe, and Yamada 1988). Several human studies suggested the presence of respiratory‐related neurons in the ventrolateral medulla. Schwarzacher, Rub, and Deller (2011) anatomically identified the location of the pre‐Bötzinger complex, well characterized as part of VRG, as a region around 5–6 mm in the ventrolateral region of the rostral medulla, 9 mm from obex, below Fissura Pontomedullaris. In LMI patients, Prabhakar et al. (2020) found that the network associated with central hypoventilation syndrome was located in the ventrolateral region of the rostral medulla in MRI. Our study further delineated that “large and typical” infarctions exhibit a higher incidence in LMI patients who subsequently develop RF. It was speculated that “large and typical” infarctions may be more likely to involve the VRG in LMI patients, ultimately leading to respiratory outcomes.

Interestingly, a significant proportion of patients with MMI also developed RF during the course of the disease. While only several case reports have previously noted this association, there have been few large‐sample investigations thus far (Hashimoto et al. 1989; Hata et al. 2014). We found that long lesions among MMI patients extending from the ventral to the dorsal medulla, particularly those involving bilateral regions and appearing as V‐shape or heart‐shape on MRI, appear to be more prone to experiencing RF during the course of the disease. Typically, these patients concurrently manifest tetraplegia, indicative of a severe clinical presentation and poor functional outcome, necessitating heightened clinical vigilance (Hu et al. 2022). Previous studies have found that the incidence of RF in patients with unilateral MMI was approximately 5%, yet almost one‐fourth of patients with bilateral MMI develop RF (Kameda et al. 2004; Pongmoragot et al. 2013). Currently, few studies have explored the underlying mechanisms of RF in bilateral MMI. It was believed that DRG was located in the ventrolateral region of the nucleus of the solitary tract (Ezure, Manabe, and Yamada 1988; Saether, Hilaire, and Monteau 1987), which anatomically corresponded to a more medial and dorsal position in the human medulla. Pattinson et al. (2009) identified respiratory‐related regions in the dorsal medulla in vivo using functional and structural imaging, with this area appearing as medial and dorsal on functional MRI. We hypothesize that long lesions extending from the ventral to the dorsal medulla were more likely to affect this region, ultimately leading to RF. Functionally, the medullary respiratory network was bilaterally innervated, suggesting that bilateral lesions may be associated with a higher risk of RF (Dyken, Afifi, and Lin‐Dyken 2012). Hata et al. (2014) reported a case of sudden death in a patient with MMI, and they demonstrated that despite autopsy findings did not indicating ischemic necrosis in the nucleus ambiguus, positive immunohistochemistry using amyloid precursor protein (APP) in neuronal tissue suggested acute ischemia in the nucleus ambiguus. We hypothesize that in MMI patients, even in the absence of radiological evidence for infarction associated with the nucleus ambiguus in the ventrolateral medulla, histopathological examination may reveal lesions impacting the neuronal networks when infarct size was considerable. Cakmakci et al. (2024) reported a case of bilateral MMI‐developed RF during the course, with MRA indicating the presence of vertebrobasilar dolichoectasia (VBD) in the patient. In our study, among 14 MMI patients concomitant with VBD, 4 exhibited bilateral lesions, with 1 patient developing RF during the course of the disease. VBD was now well known as the cause of bilateral MMI (Bhatele and Pai 2024). Bidirectional blood flow within dilated vessels can alter the morphology of the perforating arteries, resulting in reduced blood flow and thrombus formation, ultimately leading to synchronized bilateral ischemic lesions (Cakmakci et al. 2024). Antoncic et al. (2015) reported a case of bilateral MMI complicated by neurogenic pulmonary edema, leading to RF during the disease course. Whether bilateral infarctions in the medial medulla affect the “NPE trigger zones” warrants further investigation. Bilateral MMI, appearing as V‐shaped or heart‐shaped lesions on MRI, demonstrates a significant correlation with severe clinical manifestations, particularly tetraplegia, ultimately resulting in poor prognosis. Moreover, our study revealed that V‐shaped or heart‐shaped bilateral MMI exhibited a substantial association with RF, which may precipitate unexpected and potentially fatal outcomes. Therefore, greater attention should be directed toward this radiological presentation.

Furthermore, multisegment involvement was more common among LMI patients experiencing RF compared to those without. However, this difference was not observed among MMI patients. VRG was a longitudinal, strip‐like region extending from the rostral to the caudal in cats, suggesting that the medullary respiratory network was not confined to a single region but rather involved multiple segments extending from rostral to caudal (Sasaki et al. 1989). Previous observations pointed out that lesions confined to the caudal medulla were unlikely to result in RF, which may be related to the pre‐Bötzinger complex being located in the rostral medulla (Pavsic et al. 2021; Schwarzacher, Smith, and Richter 1995). In our study, we did not find rostral involvement to be a significant risk factor for RF in MI patients, despite a tendency for rostral involvement in MMI patients. This may be related to the limited number of cases included in our study. The respiratory network was complex and involved longitudinally extending groups that were interconnected via multiple interneurons and not located in a single spotted region. Pavsic et al. (2021) have suggested that extensive damage involving the open/rostral medulla appeared to be crucial for the development of RF in pure LMI. Although we identified several imaging features potentially related to respiratory outcomes, the development of RF did not appear to be associated with a single imaging pattern but rather involved a combination of infarct characteristics. Therefore, further researches were needed to explore the imaging features of MI combined with RF.

Our study demonstrated that severe bulbar symptoms were associated with respiratory outcomes in both LMI and MMI patients. It has been established that dysphagia in LMI was usually caused by direct involvement of the nucleus ambiguus, which was also an important part of the medullary respiratory network (Jang and Kim 2021). This anatomical proximity contributes to their clinical symptomatology being somewhat interrelated. Research by Kwon, Lee, and Kim (2005) found that dysarthria and dysphagia are also common and severe complications in MMI patients, which aligns with the results of our study. The bulbar symptoms in MMI patients have distinct clinical and anatomical characteristics from those in LMI patients, and the former may be related to damage to the corticobulbar tract innervating the nucleus ambiguus rather than direct involvement of this structure. We hypothesize that this pattern of involvement of the nucleus ambiguus may also affect the function of the medullary respiratory network, leading to RF in clinical course.

In addition, our study revealed an association between pulmonary infections and the occurrence of RF during the course of MI. Previous studies had reported the tendency toward severe bulbar dysfunction, particularly dysphagia, and aspiration pneumonia in patients with large rostral or middle medullary lesions (T. Kim et al. 2015). We speculated that pulmonary infections potentially acted as the triggering factor for RF. Furthermore, our study identified a correlation between urinary retention and RF in LMI and MMI patients. It was generally accepted that the pontine micturition center (PMC) in the dorsolateral pontine tegmentum serves as the central structure in micturition. However, during the micturition process, efferent fibers from the PMC descend through the lateral medulla to control micturition behavior (Cho et al. 2015). As these fibers passed near the medullary respiratory group during their descent, patients with MI may concurrently experience RF and urinary retention.

Our study did not identify significant differences in neurological symptoms/signs related to RF among patients with LMI and MMI. The two groups exhibited severe bulbar symptoms, urinary retention, and pulmonary infections correlating with RF. It was hypothesized that the histopathological locations ultimately developing RF were similar in different MIs despite variations in arterial supply and pathogenic mechanisms. Areas around the medullary respiratory network also exhibited higher risks of involvement, leading to consistent clinical manifestations. However, due to limitations in sample size, only the aforementioned three clinically significant features were identified. Other clinical manifestations, such as hiccup, which was also controlled by areas proximal to the respiratory center, did not exhibit correlation in this study, suggesting other potential mechanisms warranting further investigation.

In this study, RF in patients with MI occurred early in the course of the disease, with some patients experiencing it within 24 h of onset, consistent with most case reports (Terao et al. 2004). Overall prognosis for patients with MI complicated by RF was poor, with only approximately 15% showing favorable outcomes, while around half of the patients ultimately succumbed to mortality. Therefore, particular attention to the early course of patients with MI is imperative.

### 4.1 Limitation

Several limitations were noted in our study. First, it was a single‐center investigation. Although it included a considerable number of patients with RF, the proportion of RF cases was still relatively low compared to non‐RF cases, potentially leading to statistical biases due to insufficient sample size. Second, neurological symptoms before and after deterioration among patients who experienced RF and were promptly placed on mechanical ventilation might not have been thoroughly evaluated. Third, in our study, most of the patients experienced a short interval between the onset of RF and death, which resulted in a lack of opportunity for these patients to undergo MRI after the occurrence of RF to further elucidate the progression of infarct lesions. Consequently, the lesion patterns identified in this study were not the direct precipitants but the associated factors of RF during the disease course.

## 5 Conclusion

Our study reinforces and expands upon prior observations indicating that RF was significant complication of both LMI and MMI. Within different types of infarctions, there were specific lesion patterns, such as large and typical infarctions in LMI patients or long lesions, particularly those with bilateral infarctions, as V‐shape or heart‐shape on MRI in MMI patients, which appear to correlate with higher incidences of RF. Moreover, regardless of LMI or MMI, bulbar impairment, including severe dysphagia and dysarthria, urinary retention, and pulmonary infections, was significantly more prevalent in patients who experienced RF. The combination of MI with RF was more common in the early stages of the disease and carried poorer outcomes. Therefore, for clinicians, particular attention to these radiological and clinical features early in the disease may potentially produce favorable outcomes for patients with MI.

## Author Contributions

**Na Zhao**: conceptualization, writing–review and editing, writing–original draft, formal analysis. **Ran Liu**: data curation, resources. **Yajing Zhang**: conceptualization, writing–review and editing. **Ling Ling**: data curation, resources. **Chao Zhang**: methodology. **Ting Zhang**: conceptualization, writing–review and editing. **Wei Yue**: conceptualization, writing–review and editing.

## Ethics Statement

The study protocol received approval from the Ethics Committee of Huanhu Hospital. Given the retrospective nature of the study, the necessity for written informed consent was waived.

## Conflicts of Interest

The authors declare no conflicts of interest.

### Peer Review

The peer review history for this article is available at https://publons.com/publon/10.1002/brb3.70259.

## Data Availability

The data used to support the findings of this study are available from the corresponding author upon request (hhyuewei2021@163.com).
